# Effect of Environmental and Socioeconomic Factors on Increased Early Childhood Blood Lead Levels: A Case Study in Chicago

**DOI:** 10.3390/ijerph21040383

**Published:** 2024-03-22

**Authors:** Jangho Lee, Michael Hu

**Affiliations:** 1 Department of Earth and Environmental Sciences, University of Illinois Chicago, Chicago, IL 60607, USA; 2 PGY3 Internal Medicine-Pediatrics, School of Medicine, University of Illinois Chicago, Chicago, IL 60607, USA

**Keywords:** lead poisoning, LST, environmental justice, environmental inequality, climate change

## Abstract

This study analyzes the prevalence of elevated blood lead levels (BLLs) in children across Chicagoland zip codes from 2019 to 2021, linking them to socioeconomic, environmental, and racial factors. Wilcoxon tests and generalized additive model (GAM) regressions identified economic hardship, reflected in per capita income and unemployment rates, as a significant contributor to increased lead poisoning (LP) rates. Additionally, LP rates correlate with the average age of buildings, particularly post the 1978 lead paint ban, illustrating policy impacts on health outcomes. The study further explores the novel area of land surface temperature (LST) effects on LP, finding that higher nighttime LST, indicative of urban heat island effects, correlates with increased LP. This finding gains additional significance in the context of anthropogenic climate change. When these factors are combined with the ongoing expansion of urban territories, a significant risk exists of escalating LP rates on a global scale. Racial disparity analysis revealed that Black and Hispanic/Latino populations face higher LP rates, primarily due to unemployment and older housing. The study underscores the necessity for targeted public health strategies to address these disparities, emphasizing the need for interventions that cater to the unique challenges of these at-risk communities.

## 1. Introduction

Childhood lead poisoning is a critical public health issue as the neurotoxic effects of lead exposure pose a significant risk to the developing brains and bodies of children [1,2,3]. Blood lead levels (BLLs) in children can increase through various exposure pathways. Children can ingest or inhale lead-contaminated dust and paint chips from surfaces coated with lead-based paint [4,5,6]. Additionally, lead poisoning can occur through the consumption of tap water contaminated with lead, often due to the presence of old lead-containing water pipes [7,8,9]. Lastly, lead can be sourced from industrial sites; many neighborhoods built near these industrial sites can thus be exposed to higher BLLs [10].

Childhood lead poisoning has been a great concern, specifically in Chicago, due to the city’s extensive inventory of older housing and infrastructure, much of which predates the 1978 ban on lead-based paint. The prevalence of lead service lines supplying water to homes further exacerbates the risk of exposure. Despite ongoing efforts to mitigate these risks through public health initiatives and infrastructure improvements [11,12], the persistence of lead in the environment continues to pose a significant challenge [13,14,15,16,17,18,19,20,21].

Another factor that could contribute to elevated BLLs is the influence of climatic conditions, particularly the surface temperature. For instance, elevated temperatures can enhance the solubility and subsequent release of lead in lead-containing lines and pipes [22]. Additionally, further research, such as the study by Yiin, Rhoads [23], has shown that variations in dust lead levels peak during the summer months, aligning with the fluctuations observed in BLLs. This correlation suggests that warmer climates and seasonal temperature increases may exacerbate lead exposure risks, underlining the importance of considering environmental factors in lead poisoning prevention strategies. This consideration gains further importance in the context of climate change, driven by human activities. The interaction between a warming climate and the expansion of urban areas is likely to accelerate the rise in surface temperatures in urban locales, where more than 80% of the U.S. population resides [24,25,26]. Consequently, this phenomenon underscores the significance of increased BLLs, not only in Chicago but also in urban centers across the U.S. and globally.

Elevated BLL have emerged as a critical concern within the discourse on social and environmental justice [27,28,29,30,31]. Neighborhoods grappling with socioeconomic inequities often bear the brunt of this public health challenge, underscoring the urgent need for precise interventions and a dedicated effort to curtail lead exposure in Chicago’s most at-risk communities [13,16,19,20,32]. White and Gala [19] highlighted the connection between elevated BLLs and factors such as heightened crime rates and economic deprivation. Similarly, Tang and Carrel [20] identified an association between elevated BLLs and a range of demographic and socioeconomic variables, including education levels, the proportion of renter-occupied homes, demographic traits, and the age of housing.

Further compounding the issue, studies have indicated that elevated BLLs are intertwined with racial disparities [33,34,35], with Black and Hispanic/Latino communities experiencing a disproportionately high incidence of childhood BLLs [13]. The intersection of childhood lead exposure and subsequent adverse educational and economic outcomes [36,37,38] casts this disproportionate impact on minority populations as not only a public health crisis but also a profound social and environmental justice concern.

The objective of this study is to examine the environmental and socioeconomic determinants of elevated BLLs in children. Additionally, we analyze the racial disparities of elevated BLLs, which are influenced by varying socioeconomic statuses across different demographics. A distinguishing feature of this research is its expansive geographic coverage. Unlike other studies that are confined to the urban core of Chicago [13,39], our research encompasses the broader Chicagoland area, utilizing data at the zip code level to achieve high spatial resolution. Moreover, this study capitalizes on the latest available data, spanning from 2019 to 2021, in contrast to other research, which typically depends on data gathered prior to 2013 [13,40]. Another innovative aspect of our approach is the incorporation of land surface temperature data derived from satellite observations, a novel method in the realm of BLL research, which can have implications regarding the impact of climate change health outcomes. The inclusion of this climatic variable is intended to foster a more holistic understanding of the multiple factors influencing elevated BLLs and to supplement the existing literature with new insights and analytical dimensions.

## 2. Data

### 2.1. Blood Lead Levels (BLLs) and Lead Poisoning (LP) Data

The dataset on childhood lead poisoning was obtained from the Illinois Department of Public Health (IDPH) and represents an average from 2019 to 2021, aggregated at the zip code level as released by IDPH. It comprises self-reported information from numerous pediatric offices across the state, detailing the lead testing outcomes for more than 200,000 children each year. Our analysis focuses on the percentage of the children’s population with BLLs over 5 µg/dL, a threshold previously established by the Centers for Disease Control and Prevention (CDC) as a “reference level” at which children should be monitored [15]. This metric, a prevalence rate of children with over 5 µg/dL of BLL, will be referred to as the lead poisoning (LP) rate hereafter. 

**Figure 1:**
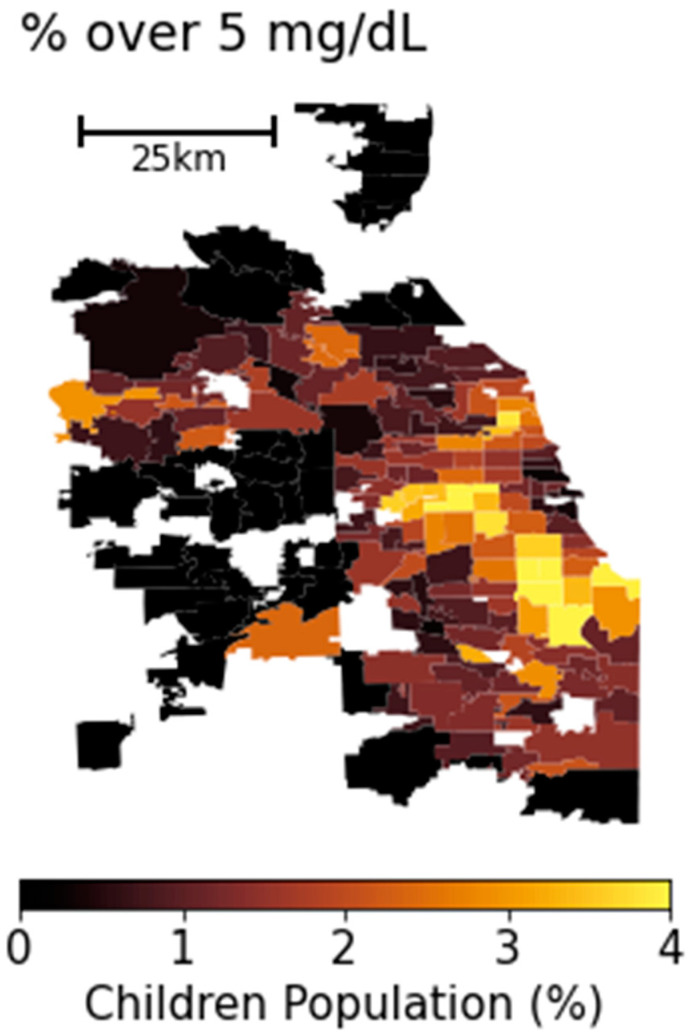
Map of percentage of children with over 5 µg/dL of BLLs (LP) in Chicago area for 2019–2021 period, aggregated at the zip code level.

A significant advantage of these data is their coverage, stemming from the state of Illinois’ mandates that healthcare providers test children for lead exposure during office visits. Children are required to be tested at 12, 24, and 36 months of age, ensuring comprehensive data collection. Figure 1 presents a map of LP rate, analyzed at the zip code level. Other metrics, other than5 µg/dL of BLL, can be used as a proxy for LP rate. However, the correlation coefficient between Figure 1 and another map, where the threshold is set at 3.5 µg/dL, is 0.87. This high degree of correlation suggests that analyses based on either threshold are likely to yield similar results. We used a total of 181 zip codes in this study, with an average LP rate of 1.36%.

### 2.2. Socioeconomic Data

**Figure 2:**
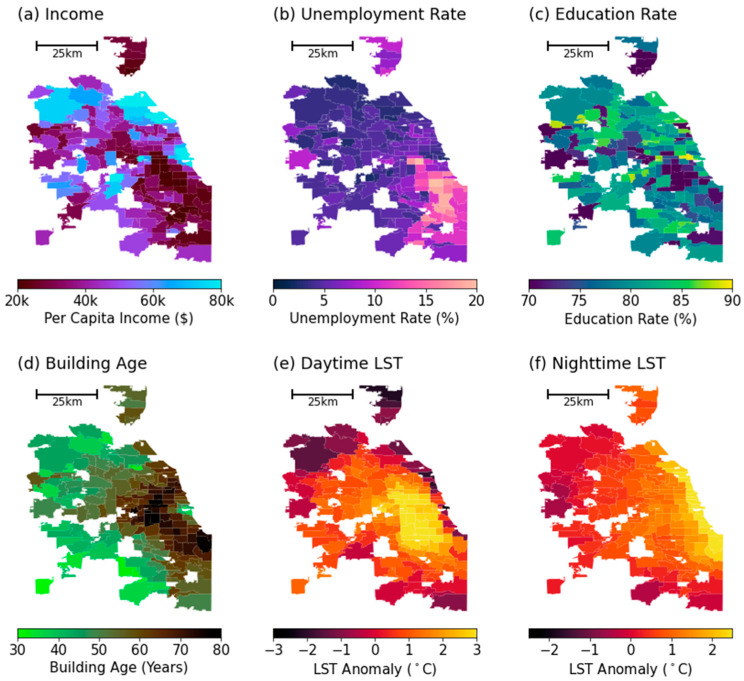
Zip-code-level map of socioeconomic variables and land surface temperature metrics. (**a**) Average per capita income (income), (**b**) unemployment rate (unemployment), (**c**) percentage of population with a high school diploma (education), (**d**) years after the building was built (building age), (**e**) average daytime (10:00~15:00) LST anomaly for summertime (June, July, and August) from 2018 to 2021, and (**f**) average nighttime (00:00~05:00) LST anomaly for summertime from 2018 to 2021.

The socioeconomic data employed in this analysis were sourced from the U.S. Census Bureau, concentrating on zip-code-level metrics including average per capita income, unemployment rate, education rate (the percentage of individuals with a high school diploma), and the average age of buildings (calculated as the average number of years since construction). This compilation represents a five-year average for the interval between 2017 and 2021. The socioeconomic indicators are graphically depicted in Figure 2a–d.

The selection of these variables was driven by several considerations. Average per capita income and unemployment rates serve as vital economic indicators, reflective of a community’s economic health and stability, factors that can indirectly impact environmental health risks such as lead exposure [27,41,42,43]. The education rate is employed as an inverse measure of social vulnerability, with the premise that higher educational levels might be associated with lower risks of exposure and a greater capacity for advocacy in environmental health matters [44]. The inclusion of building age assumes that older buildings are more probable repositories of lead-based paints and plumbing, thus representing a more direct risk factor for lead exposure [10,12,45,46]. Together, these variables provide a robust framework to dissect the intricate relationship between socioeconomic factors and environmental health hazards, especially the risk of high LP rates across varied communities.

### 2.3. Land Surface Temperature Data

In addition to socioeconomic data, this study uniquely incorporates land surface temperature (LST) estimates from the GOES-16/17 satellites [47,48], which provide continuous, hourly LST readings across North America at a spatial resolution of 2 km. These high-resolution, temporally dense LST measurements are appropriate for zip-code-level analyses, enabling a thorough investigation of regional LST variations and their potential connections to LP rates. This level of detail is crucial, as alternative temperature estimates, such as station-based data, lack the spatial resolution necessary to capture the details of intracity temperature variations. Station-based measurements are often too sparsely situated to accurately reflect the intricate patterns of urban temperature dynamics. For example, within the Chicagoland area, there are only nine weather stations recording temperature data according to the National Centers for Environmental Information (NCEI). This limited number is insufficient to discern the complex temperature gradients present within the urban area, underscoring the value of satellite-derived LST data in providing a more granular and comprehensive perspective of urban thermal landscapes [49].

An inherent limitation of satellite-measured LST products is their inability to capture surface temperatures under cloudy conditions. To address this limitation, we refined our dataset by exclusively selecting observations recorded during hours with less than 10% cloud cover within our study area. This selection criterion ensures that we utilized a subset of data that can be reliably considered cloud-free. As a result of this filtering process, we obtained 7724 h of usable LST data from 2019 to 2021, constituting approximately 30% of the total available temperature data. This subset is adequate for our analysis as our primary interest lies in examining the relative temperature differences across zip codes rather than the absolute magnitude of LST.

As a secondary step in our analysis, we calculated the LST anomaly for each of the 7724 time steps. For each grid point within the 2 km resolution data, we subtracted the mean LST of the entire study area at the corresponding time step to determine relative temperature differences. This calculation produced 7724 spatial anomaly maps.

Considering the distinctive thermal dynamics in urban environments, where the surface urban heat island (SUHI) effect leads to different temperature patterns during day and night [50,51,52], we calculated two key metrics from these anomaly maps: the daytime LST anomaly and the nighttime LST anomaly. The daytime LST anomaly is the average anomaly determined from time steps between 10 am and 3 pm local time, reflecting influences such as solar radiation and vegetation cover. The nighttime LST anomaly, in contrast, is computed using data from midnight to 5 am local time, capturing the thermal inertia of urban structures [53,54,55,56,57].

Finally, we aggregated the 2 km gridded daytime LST anomaly and nighttime LST anomaly maps to the zip code level to align with the granularity of LP data and socioeconomic variables. For each zip code, we averaged the anomalies of grid points within its boundaries to derive zip-code-level daytime and nighttime LST anomaly values. These aggregated values yield two comprehensive spatial maps depicting daytime and nighttime LST anomalies for the Chicagoland area, illustrated in Figure 2e,f. Moving forward in our discussion and analysis, the terms ‘daytime LST anomaly’ and ‘nighttime LST anomaly’ will be simplified to ‘daytime LST’ and ‘nighttime LST’, respectively.

### 2.4. Interrelationships among Predictors

**Figure 3:**
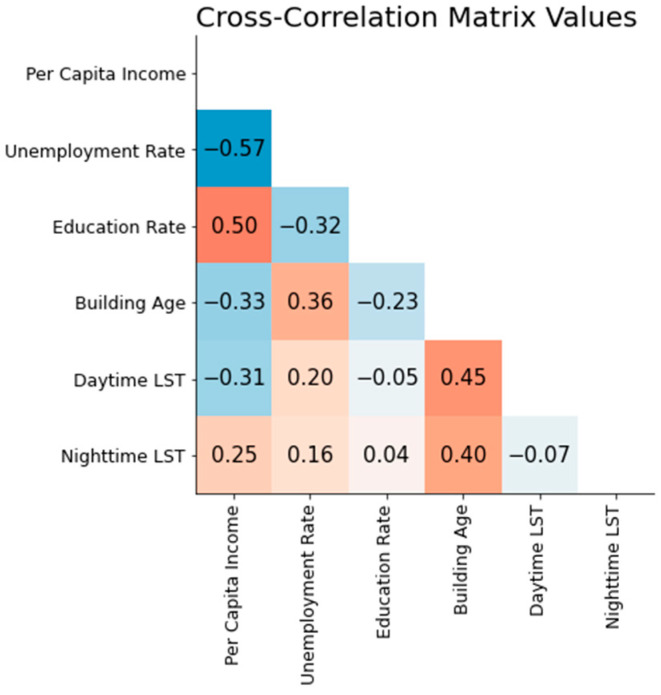
Heatmap displaying the cross-correlation matrix of predictor variables with correlation coefficients.

Assessing the interrelationships among predictor variables is a critical step in ensuring the robustness of our analysis. Figure 3 illustrates the cross-correlation matrix for the predictor variables, providing insights into the potential multicollinearity that could affect the interpretation of the results. The cross-correlation analysis indicates that the highest correlation exists between the unemployment rate and per capita income, with a coefficient of −0.57. While this is substantial, it does not preclude the inclusion of both variables in our model, as the inclusion of both enhances our model’s performance. 

## 3. Method of Analysis

In this study, we utilized the generalized additive model (GAM) as the regression framework to estimate the impact of each predictor variable (per capita income, unemployment rate, education rate, building age, daytime LST, nighttime LST) on LP rate. GAM extend the capabilities of linear regression models by accommodating non-linear relationships between the predictors and the response variable, offering a more detailed approach to data analysis [58]. In contrast to traditional linear or polynomial regression, which assumes linear or fixed connections among variables, the GAM leverages multiple cubic splines to generate smooth functions to model these relationships, providing a flexible methodology that aligns with the actual data distribution.

In our approach, a spline function is fitted for each predictor variable, utilizing a basis of 12 cubic splines. This configuration allows the GAM to produce a smooth curve that accurately reflects the relationship between each predictor and the dependent variable. Opting for 12 cubic splines grants the model ample flexibility to capture complex, non-linear patterns effectively. To enhance the fidelity and generalization ability of these spline functions, smoothing parameter (λ) values are meticulously adjusted for each predictor. These λ values play a pivotal role in modulating the smoothness of the spline functions. To determine the optimal λ values, we employed a grid search algorithm by testing a spectrum of λ values to pinpoint the one that minimizes the root mean squared error (RMSE) of the model’s predictions, thereby ensuring a precise and reliable prediction of LP rate.

In this study, the Python libraries pyGAM [59], SciPy [60], GeoPandas [61], and matplotlib [62] were employed to perform statistical analyses and generate figures.

## 4. Results

### 4.1. Wilcoxon Signed-Rank Test for Individual Predictors

**Figure 4:**
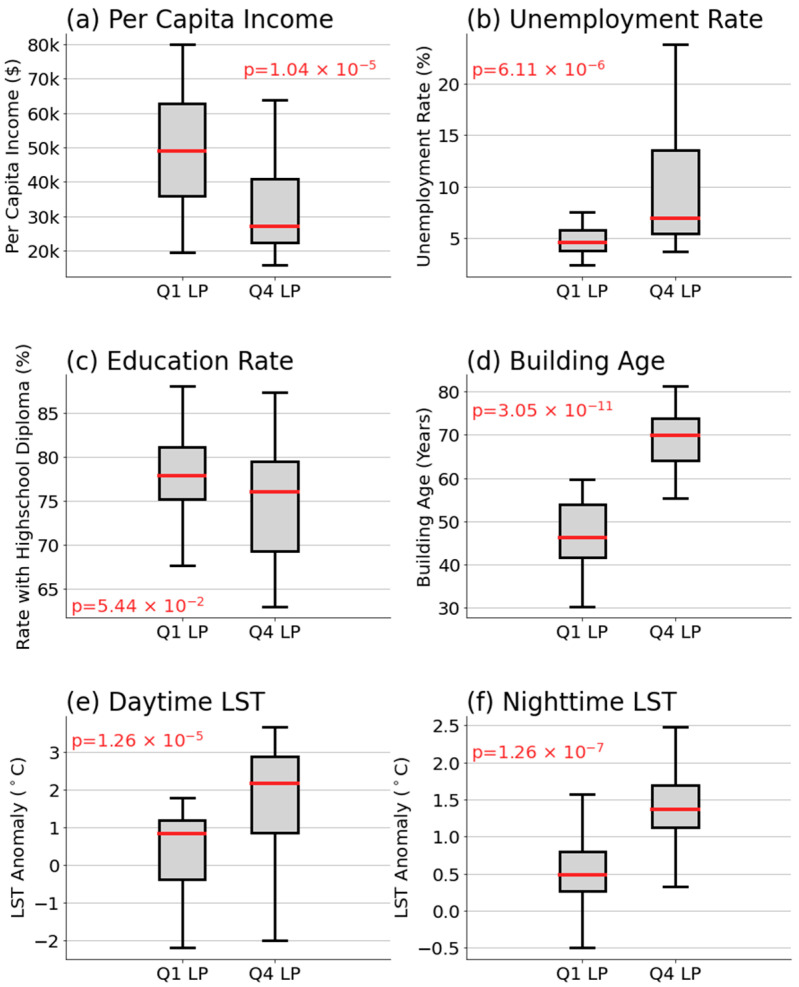
Comparative distribution of predictor variables between Q1 (left) and Q4 (right) LP rate percentile groups. The variables include (**a**) per capita income, (**b**) unemployment rate, (**c**) education rate, (**d**) building age, (**e**) daytime LST, and (**f**) nighttime LST. Within each box plot, the median values are indicated by red lines, while the boxes represent the interquartile range (IQR), spanning from the 25th to the 75th percentile. The whiskers extend to 1.5 times the IQR.

To begin our analysis, we conducted a Wilcoxon signed-rank test (hereafter, the Wilcoxon test) to examine the impact of each predictor variable on LP rate. The Wilcoxon test is a non-parametric rank test to compare the differences between two groups of samples. We categorized zip codes into two groups based on their LP rate percentiles: those falling below the 25th percentile (Q1) and those above the 75th percentile (Q4) of LP rate. We then compiled the values of each of the six predictor variables for the zip codes within these two LP rate groups. A Wilcoxon test was performed to determine if there are statistically significant differences in the predictor variables between the Q1 and Q4 LP rate groups. The distributions of predictor variables for the Q1 and Q4 groups, along with their corresponding *p*-values, are presented in Figure 4.

As depicted in Figure 4, per capita income appears to have a negative impact on LP rates (Figure 4a), suggesting that zip codes with higher LP rates tend to exhibit lower income levels. In contrast, the unemployment rate (Figure 4b) and building age (Figure 4d) have a positive impact, indicating that zip codes with higher LP rates are associated with higher unemployment rates and older buildings. Furthermore, both daytime and nighttime LST demonstrate a positive impact (Figure 4e,f), implying that regions with higher LP rates are likely to experience higher surface temperatures. It is notable that the education rate does not exhibit a significant discrepancy between the Q1 and Q4 groups, with a *p*-value of 0.054, which is above the conventional threshold (0.05) for statistical significance.

Per capita income and unemployment rate shows that areas that are economically underrepresented are more exposed to the risk of elevated BLLs, consistent with previous studies [10,16,29,42]. This is because these areas might also be situated in closer proximity to industrial sites or in environments with inadequate municipal services, both of which can contribute to higher lead exposure. Furthermore, limited economic resources can restrict access to health education and nutritional options that mitigate lead absorption, such as diets high in calcium and iron [63,64,65]. Lower income levels are also associated with reduced access to healthcare services, which can delay the diagnosis and treatment of lead exposure.

The strong correlation between the age of buildings and high LP rates within zip codes can be attributed to several key factors primarily associated with the construction practices and materials used in older structures, as described in previous studies [11,12,45,46]. Older buildings, particularly those constructed before the 1978 U.S. ban on lead-based paint, are likely to contain lead in their paint, pipes, and even the soil surrounding the property. As these materials age and deteriorate, lead can be released into the environment, posing significant exposure risks, especially to children. In addition to lead-based paint, older plumbing systems may contain lead pipes, solder, or fixtures that contribute to lead contamination in water supplies. As these materials corrode over time, they can leach lead into the water, increasing the risk of ingestion. Furthermore, the general wear and tear on older properties can result in chipped or peeling paint, which can be ingested or inhaled as dust, a common exposure pathway for lead.

Lastly, elevated daytime and nighttime LST can exacerbate LP rates through a couple of mechanisms. Higher temperatures can increase the solubility of lead in old pipes, enhancing the likelihood of lead leaching into drinking water, especially in areas with aging infrastructure. As temperatures rise, the rate of chemical reactions can also accelerate, potentially increasing the rate at which lead enters the water supply [22]. Furthermore, the strong correlation coefficients between general daytime and nighttime LST and their summertime equivalents—0.98 and 0.93, respectively—indicate that the higher temperature readings are consistent during the warmer months of June, July, and August. During warmer seasons, residents are more likely to open windows to alleviate indoor heat, which can inadvertently facilitate the ingress of lead-contaminated dust from the outside environment into homes, particularly in urban areas where soil and airborne lead particles may be more prevalent due to historical emissions from gasoline, industrial activities, or deteriorating exterior lead-based paint [23]. This route of lead exposure could disproportionately affect economically disadvantaged groups, who might rely more on natural ventilation because they cannot afford air-conditioning costs.

### 4.2. LP Rate Modelling with GAM

Now, we apply GAM to estimate zip-code-level LP rates in more detail, utilizing the approach detailed in Section 3. By fitting the model with zip-code-level predictor variables, we aim to accurately estimate LP rates. The model demonstrates a strong prediction performance, evidenced by a correlation coefficient of 0.82 and a root mean squared error (RMSE) of 0.26, indicating a robust fit to the data.

**Figure 5:**
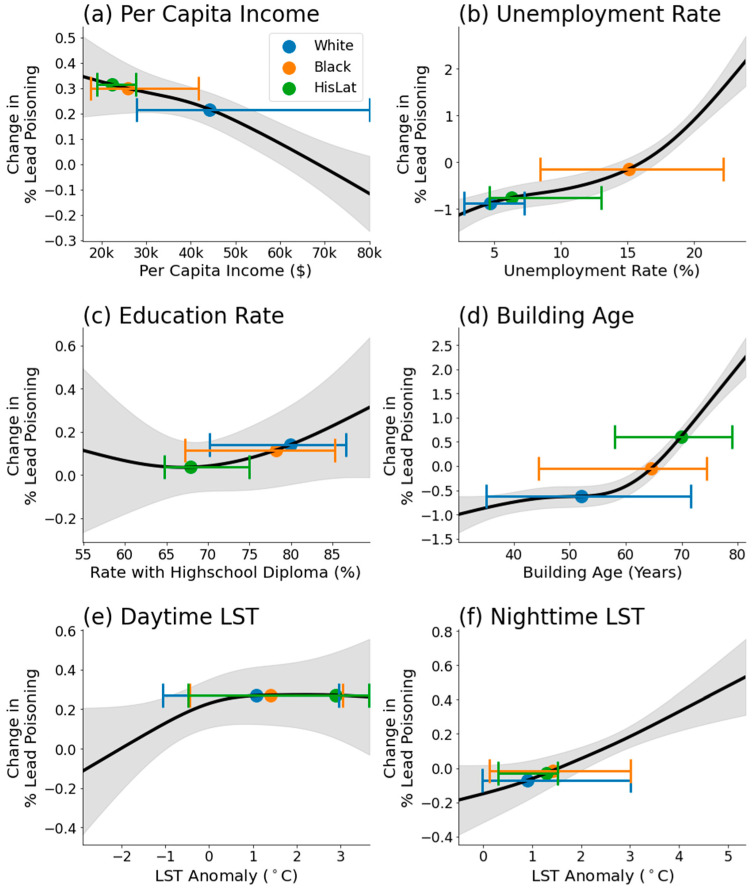
Partial dependence of each predictor variable, derived from GAM regression. The variables include (**a**) per capita income, (**b**) unemployment rate, (**c**) education rate, (**d**) building age, (**e**) daytime LST, and (**f**) nighttime LST. The black line represents the estimated partial dependence, while the gray shaded area shows the 90% confidence interval of the dependence. Blue, orange, and green dots represent the median values for each racial group, while the corresponding error bars denote the 5–95th percentile interval of predictor variables, for each racial group.

One of the significant advantages of using GAM is its ability to elucidate and depict the nature of the relationship between each predictor variable and the LP rates, regardless of whether these relationships are linear or nonlinear. This is achieved through the computation of partial dependence plots, which provide a graphical representation of the marginal effect of each predictor on the response variable. Figure 5 illustrates these partial dependence plots for each predictor variable, offering insights into how variations in income, unemployment rate, education rate, building age, daytime LST, and nighttime LST independently correlate with LP rates across different zip codes.

The outcomes of the GAM regression are consistent with the initial findings from the Wilcoxon test analysis, indicating the consistent impacts of per capita income, unemployment rate, building age, daytime LST, and nighttime LST on LP, while the education rate does not exhibit a significant effect, as evidenced by the considerable range of uncertainty depicted in the gray shaded area of Figure 5c.

Unique insights emerge from the GAM analysis, particularly concerning the non-linear impact of building age. Figure 5d illustrates a marked increase in LP rates when the average building age exceeds approximately 55 years. This observation aligns with historical context, given that lead-based paint was prohibited in 1978, approximately 45 years prior to the current analysis period. This provides a temporal benchmark, highlighting a significant increase in LP rate associated with buildings predating this ban.

The analysis of daytime and nighttime LST reveals detailed differences in their relationships with LP. While both temperatures correlate positively with LP, the influence of daytime LST plateaus after a 1 °C anomaly, suggesting a threshold effect. Conversely, nighttime LST demonstrates a steady positive relationship with LP, with higher confidence levels than its daytime counterpart. This distinction may be attributed to the better representation of urban fabric by nighttime LST, which captures the retained heat within built environments more effectively than daytime LST.

Further analysis incorporating zip-code-level normalized differential vegetation index (NDVI) and normalized differential built-up index (NDBI) from Sentinel-2 satellite imagery [66]—serving as proxies for vegetation and built-up areas, respectively—reveals that nighttime LST correlates more strongly with these indices (−0.76 with NDVI and 0.61 with NDBI) compared to daytime LST (−0.31 with NDVI and 0.44 with NDBI). Given the relatively small geographical extent of the Chicagoland area, which suggests minimal large-scale climatic variation across the region, these findings underscore the premise that nighttime LST, as a more accurate indicator of urban temperature dynamics, holds greater significance in this analysis.

### 4.3. Racial Inequality

Until this point, the analysis has concentrated on per capita income, unemployment rate, building age, and LST, without integrating racial demographics for each block. This approach is because, within the Chicagoland context, racial demographics exhibit a high correlation with the aforementioned socioeconomic and environmental metrics, suggesting that including racial data directly does not significantly enhance the predictive capability of our model. Nonetheless, the GAM framework allows for an exploration of how LP rates may be differentially influenced by socioeconomic status across various racial groups within zip codes.

To investigate the influence of socioeconomic factors on LP rates within different racial groups, we initially identified zip codes predominantly inhabited by specific racial demographics—White, Black, and Hispanic/Latino (HisLat)—based on the criterion that the majority population (over 50%) of the zip code belongs to one of these racial categories according to the Census data. Subsequently, we compiled the values of each predictor variable (per capita income, unemployment rate, education rate, building age, daytime LST, and nighttime LST) for the zip codes categorized by these racial demographics.

For each racial group’s set of zip codes, we calculated the median values of the predictor variables to provide a central tendency measure. To illustrate the variability within each group, we also determined the 5–95th percentile range for these variables. These statistical measures are visually represented in Figure 5, where the median values are indicated by dots, and the error bars depict the 5–95th percentile ranges. This visualization offers a comparative perspective of how each racial group’s socioeconomic and environmental conditions—potentially influencing LP rates—vary within their respective, predominantly inhabited zip codes.

**Figure 6:**
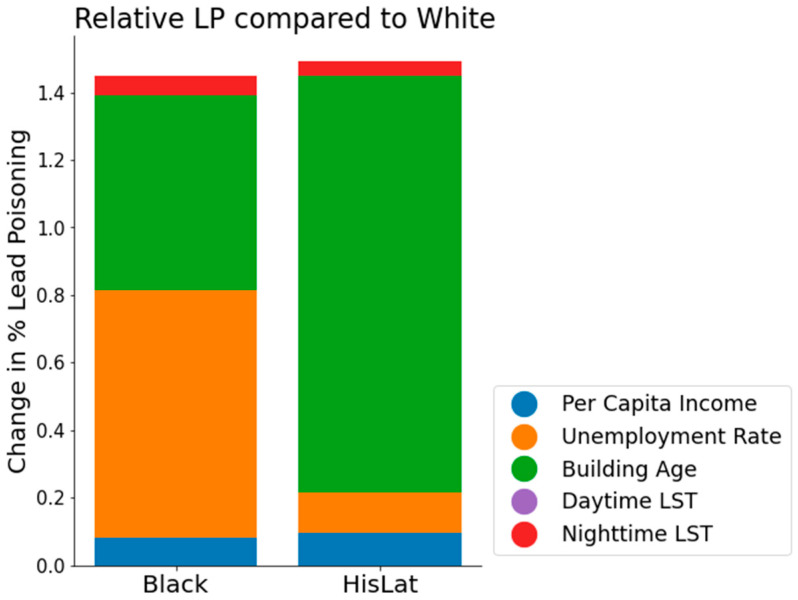
Bar graph showing the relative change in LP rates for Black and Hispanic/Latino (HisLat) populations in comparison to the White population. Each bar is segmented to demonstrate the contribution of different predictor variables: per capita income (blue), unemployment rate (orange), building age (green), daytime LST (purple), and nighttime LST (red).

By utilizing the partial dependence function provided by GAM, we gained insights into the specific impact of each predictor variable on LP rate across racially defined zip code groups. Figure 6 illustrates the comparative LP rates for Black and HisLat populations relative to the White population. Education rate is not included in this analysis since both Wilcoxon test and GAM regression did not show a significant statistical relationship with education rate and LP rate. 

As depicted in Figure 6, the Hispanic/Latino (HisLat) population exhibits the highest relative risk of LP, with a 1.49% increase in LP rate compared to the White population. The Black population shows a 1.45% increase in LP rate compared to the White population. This is a significant increase in LP rate, considering that the 5–95th percentile range of the observed LP rate is 0–3.8% in all zip codes (Figure 1). 

The underlying factors contributing to this disparity differ between the two groups. For the HisLat population, the majority of the increased LP rate is attributable to building age, which accounts for 82% of the HisLat-specific increase and 1.23% absolute increase in LP rate. In contrast, for the Black population, the increased LP rate is largely due to both the unemployment rate—contributing to 50% of the Black-specific increase and a 0.73% increase in LP rate—and building age, which accounts for 40% of the increase and a 0.58% absolute rise in LP rate.

While our modelling-based analysis identifies socioeconomic factors and building age as key contributors to LP rate disparities, it is essential to recognize these factors within a broader historical and social context. Historical housing policies, particularly redlining, have ingrained systemic racial discrimination into urban landscapes, influencing residential patterns and health outcomes to this day. These policies have not only marginalized certain communities through economic constraints but have also exposed them to greater environmental risks, including deteriorated housing and proximity to pollutants.

## 5. Summary and Conclusions

In this study, we investigated the percentage of children with blood lead levels (BLLs) exceeding 5 µg/dL across zip codes in the Chicagoland area from 2019 to 2021, examining the association with socioeconomic and environmental factors and the broader context of racial inequality.

Firstly, using Wilcoxon tests and GAM regressions, we identified economic hardship—indicated by per capita income and unemployment rate—as a factor that increases LP. The underlying mechanisms are multifaceted: economically strained areas are likely to be situated closer to industrial sites or in locales with inadequate municipal services. Additionally, limited financial resources can impede access to health education and nutrition that may prevent lead absorption, a finding supported by several previous studies [10,16,29,42,63,64,65]. 

Secondly, we established a correlation between the average age of buildings and LP rates. This finding aligns with prior research that has demonstrated a higher LP rate associated with older buildings [11,12,45,46]. Using GAM, we observed a pronounced increase in LP rate when the average building age exceeded 55 years, following the timeline of the 1978 ban on lead paint in the United States. This result not only underscores the persistent impact of lead-based paint in older buildings but also illustrates the direct influence of policy measures on health outcomes.

Moreover, our study analyzes the relationship between satellite-based measurements of land surface temperature (LST) variations during daytime and nighttime and their impact on LP rate, a previously unexplored area. We hypothesize that higher LSTs may contribute to increased LP rates by enhancing the solubility of lead in antiquated plumbing systems, thus elevating the potential for lead to leach into the drinking water supply within communities living within an older infrastructure. Our findings indicate that both daytime and nighttime LST are associated with increased LP, with a more marked effect observed for nighttime LST. The heightened impact of nighttime LST likely stems from its effectiveness as an indicator of the urban heat island effect, which captures the thermal retention properties of urban structures and surfaces. Nighttime LST is particularly indicative of the heat generated and retained by built environments, which can have various indirect effects on LP. For instance, higher nighttime LST may be reflective of less vegetated areas or denser building materials, both of which are factors that can indirectly contribute to elevated LP rate. This aspect of the study not only extends the understanding of environmental influences on LP rates but also underscores the potential of satellite-derived LST data as a valuable tool in public health research.

Furthermore, the association between increased LP rates and high LST identified in this study is a significant contribution to the area of climate change and public health. Numerous previous studies have addressed the repercussions of rising temperatures on health outcomes, focusing on aspects such as temperature-related mortality [67,68,69], the spread of vector-borne diseases [70,71,72,73], mental health [74,75,76,77], and respiratory conditions [78,79,80,81]. However, this research stands out by establishing a novel link between elevated temperatures and heightened LP rates. Given the ongoing effects of climate change and the anticipated exacerbation of urban temperatures due to the urban heat island effect—coupled with the fact that urban settings are primary sources of elevated LP rates—this discovery underscores the potential for climate change to amplify LP rates, not only in Chicago but in urban centers worldwide.

Lastly, our investigation addresses racial disparities in LP rates. We find that Black and Hispanic/Latino communities are at risk from an elevated LP rate. The factors contributing to this increased exposure differ somewhat between groups. For the Black population, the heightened risk is primarily due to the high unemployment rates and the prevalence of older housing. In contrast, for Hispanic/Latino communities, the risk is predominantly associated with the presence of older housing stock. This analysis underscores a critical public health challenge: socioeconomically disadvantaged communities, particularly Black and Hispanic/Latino populations, are disproportionately affected by LP. The compounded issue of higher LP rates and limited access to mitigating resources highlights the need for dedicated public health initiatives. There is evident demand for targeted interventions and supportive measures that are tailored to address the distinct challenges faced by these vulnerable groups. Such initiatives are essential not only to alleviate the immediate burden of LP but also to foster long-term health and wellbeing within these communities. Also, the observed higher rates LP within minority populations merit attention, particularly considering how stressors associated with poverty, racism, and stereotype threats prevalent among these groups could influence the body’s biotransformation processes. Such factors might contribute to the disparities in LP rates observed across different demographic groups.

This study is notable for several reasons. Firstly, it utilizes recent data from 2019 to 2021, providing a contemporary analysis of the factors contributing to LP. Secondly, it extends beyond the urban center to encompass the wider Chicagoland area, offering insights into a broader demographic. Thirdly, satellite-based measurements of LST are employed, highlighting the value of remotely sensed environmental data in public health research and further highlighting the possible increase in LP in a warming world. Fourthly, advanced statistical modeling through GAM is applied to understand the non-linear effects of socioeconomic and environmental variables on LP rates. Lastly, the study addresses racial disparities by examining how socioeconomic conditions correlated with race indirectly affect LP rates, providing an informed perspective on this complex issue.

## 6. Limitation and Future Direction

Despite its strengths, the study is not without limitations. Although this study has several strengths, it is not without its limitations. Firstly, the study did not consider various neighborhood environmental factors beyond building age. For instance, it does not account for the presence of Comprehensive Environmental Response, Compensation, and Liability Information System (CERCLIS)-listed sites in the vicinity, which may contain hazardous substances such as heavy metals, including lead [82].

It is important to acknowledge that while our regression model did not reveal a significant correlation between LP and educational factors, this could be due to the high correlation between education levels and other variables, such as income and unemployment rates, which have demonstrated strong associations with LP. Additionally, the lack of observed significance may be attributed to the spatial and temporal scales considered in this study.

Furthermore, the dataset covers a relatively short period of just three years. With a more extended dataset, it would be possible to examine the general trends or zip-code-specific trends in LP rates over time, assessing not just the disparities between zip codes but also how they evolve. Additionally, other environmental factors, such as wind speed or humidity, could potentially enhance the analysis. However, obtaining these variables at a high spatial resolution is challenging, as they are not readily measurable via satellite technologies.

## Data Availability

Socioeconomic and ethnic data for Chicago region can be accessed in U.S. Census bureau website (https://data.census.gov/ (accessed on 15 January 2024)). GOES-16 and 17 data are available on Amazon Web Service (AWS) S3 Explorer GOES-16: https://noaa-goes16.s3.amazonaws.com/index.html (accessed on 15 January 2024); GOES-17: https://noaa-goes17.s3.amazonaws.com/index.html (accessed on 15 January 2024). Sentinel-2 imagery used to calculate NDVI and NDBI are from Google Earth Engine (https://developers.google.com/earth-engine/datasets/catalog/sentinel-2 (accessed on 15 January 2024)). Zip code level BLL and LP rate data are from Illinois Department of Public Health (IDPH).
